# Telemedicine Consultation in Neonatal Resuscitation: A Randomized Crossover Simulation Study

**DOI:** 10.1159/neo/adsag002

**Published:** 2026-08-26

**Authors:** Andrea Lietz, Andreas Goreis, Michael Wagner, Antonia Lair, Tereza Schedler, Stefano Mahlberg, Oswald D. Kothgassner, Angelika Berger, Judith Rittenschober-Boehm

**Affiliations:** a Division of Neonatology, Department of Pediatrics and Adolescent Medicine, Pediatric Intensive Care and Neuropediatrics, Comprehensive Center for Pediatrics, Medical University Vienna, Vienna, Austria; b Department of Child and Adolescent Psychiatry, Medical University Vienna, Vienna, Austria; c Department of Neonatology, Kepler University Hospital, Linz, Austria

**Keywords:** Augmented reality, Neonatal intensive care unit, Neonatal life support, Telemedicine

## Abstract

**Introduction:**

Telemedicine consultation can provide real-time expert support to local teams during neonatal emergencies. This study aimed to assess the impact of telemedicine support on provider performance and user experience during simulated neonatal resuscitation, using three devices of differing technical complexity.

**Methods:**

In this randomized crossover study, 93 medical students and doctors completed two neonatal resuscitation scenarios – one without and one with telemedicine support – using one of three randomly assigned devices: a phone, a video telemedicine device, or augmented reality (AR) glasses. The primary outcome, neonatal life support (NLS) performance, was assessed using a 33-item checklist based on a standardized NLS score valuated by two independent raters. Secondary outcomes included user experience, device usability, and participants’ gain of performance by using telemedicine support, stratified by participants’ level of training.

**Results:**

Resuscitation performance improved significantly with telemedicine support compared to the control scenario (38.37 vs. 33.02 points, *p* < 0.001), with no difference between the three telemedicine conditions. In the scenario without telemedicine, performance was significantly associated with participant training level, while telemedicine mitigated this effect, yielding the greatest performance gains among untrained users (*r* = −0.34, *p* < 0.001). User experience ratings were the lowest for AR glasses, primarily due to technical issues. Less trained participants rated telemedicine as more helpful and beneficial on simulation outcomes.

**Conclusion:**

This simulation-based pilot study demonstrates that telemedicine consultation can significantly improve neonatal resuscitation performance, especially among less trained providers. Differences in user experience underscore that “simple-to-use” technology is needed by healthcare providers when using telemedicine.

## Introduction

Infant mortality is the highest on the first day of life [1]. Although most newborns transition without complications, approximately 5% require immediate respiratory support and up to 0.3% need extensive resuscitation [2]. This underscores the importance of skilled neonatal care during this critical period, which may be limited in remote hospitals [3]. In the USA, 15–20% of very low birth weight infants and 45–60% of infants with hypoxic-ischemic encephalopathy are born in hospitals without a Neonatal Intensive Care Unit [4–7]. This “outborn” status is associated with higher mortality and morbidity [4, 5]. Telemedicine enables neonatologists from perinatal centers to immediately provide real-time support to the local care team [4–6, 8]. Previous studies have suggested that telemedicine can improve the quality of care, reduce unnecessary transfers, and potentially lower healthcare costs, with high satisfaction reported by both, community hospital staff and remote neonatologists [9–12]. Accordingly, the recently published European Resuscitation Council Guidelines 2025 recommend considering the use of telemedicine, yet evidence remains limited [12].

Effective telemedicine implementation requires identifying the most appropriate device for the clinical context. While most teleconsultation studies have utilized basic video calls, advanced technologies such as telehealth stations or mixed-reality glasses are emerging, despite reported technical challenges. Consequently, identifying the optimal platform for neonatal teleconsultation is essential [8]. This study aimed to assess the impact of telemedicine support on provider performance and experience during simulated neonatal resuscitation, using three telemedicine devices with differing technical complexity.

## Methods

### Study Design

This randomized crossover simulation study was conducted during May 2023 and January 2024 at the Pediatric Simulation Training Center, Medical University of Vienna, Austria. Participants led a simulated neonatal resuscitation scenario (online suppl. Material, SM1; for all online suppl. material, see https://doi.org/10.1159/neo/adsag002) twice – with and without remote support from a neonatal consultant. Randomization was generated using www.randomizer.org. Participants were first randomized 1:1 to perform the initial scenario either with or without telemedicine support and subsequently assigned to one of three telemedicine devices. The study design is illustrated in Figure 1. Demographic data were collected via questionnaires.

**Fig. 1.**
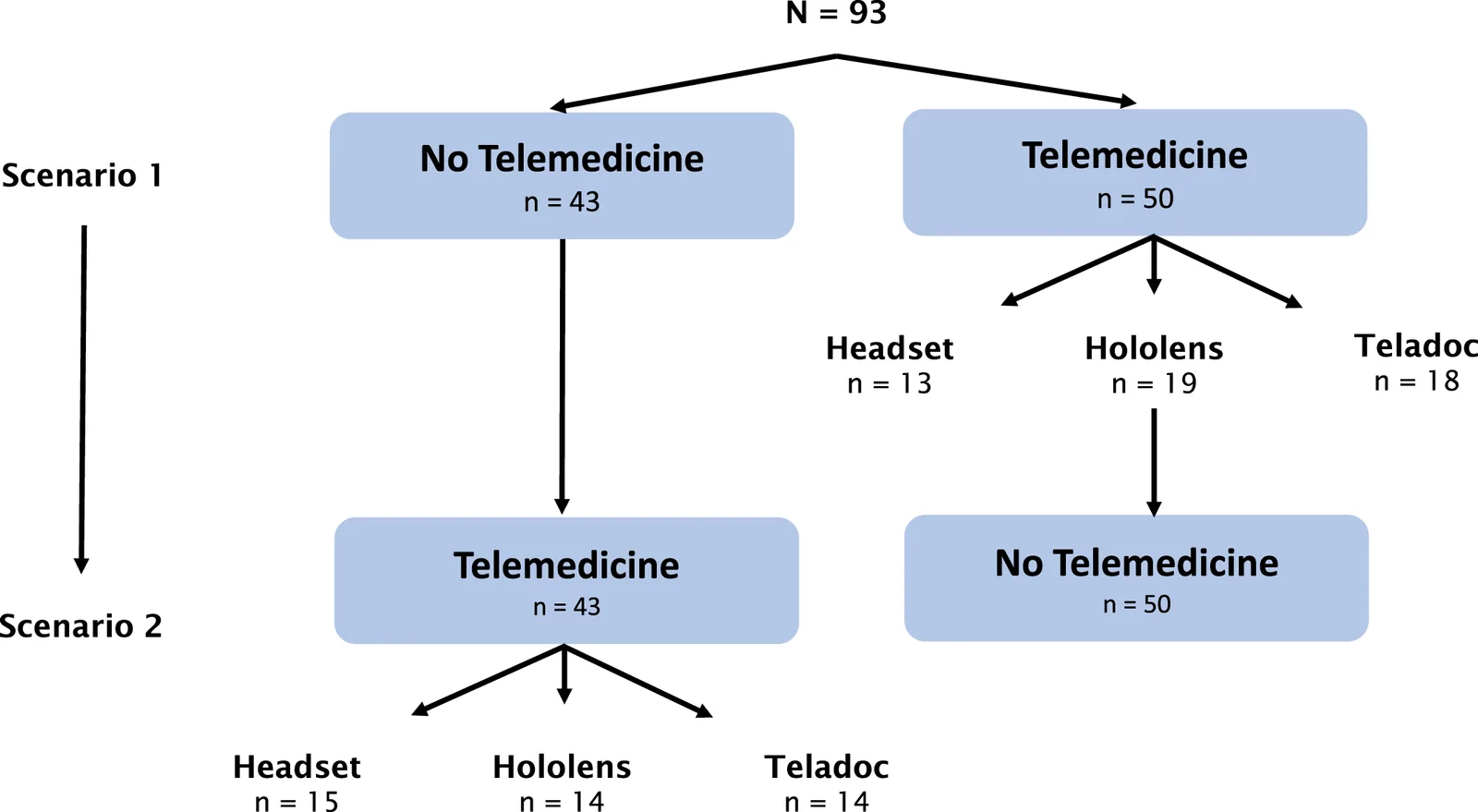
Schematic overview of the study design. Participants were randomized to the sequence of simulation conditions, starting either with or without telemedicine support, and to one of three telemedicine devices for the supported scenario. All simulations with telemedicine involved the placental abruption scenario, whereas all simulations without telemedicine involved the shoulder dystocia scenario. The scenarios were of comparable complexity.

### Participants

Medical students and doctors with various levels of training in the fields of neonatology and anesthesiology were eligible to participate. Participants were recruited voluntarily through departmental postings or email invitations and registered on a first-come, first-serve basis via a provided link. Based on participants’ training background, they were assigned to a “level of training” ranging from 1 to 15 (online suppl. Material SM2). A single highly experienced neonatal consultant (level of training: 15) served as the remote expert for all participants to minimize variability in consultation. After registration, participants received a teaching video demonstrating the latest guidelines for neonatal resuscitation, ensuring basic knowledge of neonatal life support (NLS) [2].

### Sample Size

Sample size was calculated using G*Power software, which indicated that a sample of *N* = 90 was required to achieve sufficient power. Details are given in online supplemental Material SM3 [13–15].

### Outcome Measures and Scoring

The primary outcome was the NLS performance, analyzed according to the randomization group. Adherence to the NLS guidelines was assessed by two independent observers (1 doctor and 1 sim-nurse, both working at the Pediatric Simulation Center), using a standardized NLS score [16]. For the present study, the checklist was adapted to the specific simulation scenarios by excluding non-applicable items, resulting in 33 items (online suppl. Material SM4) [2]. Most of the questions are dichotomous (performed/not performed), a few require multiple categories (performed/partly performed/not performed), resulting in a maximum score of 42 points. Interrater reliability for the performance score was assessed using an intraclass correlation coefficient and was good, ICC = 0.83, 95% CI [0.77, 0.87], *p* < 0.001.

The secondary outcome was user experience and usability. Participants completed questionnaires assessing satisfaction with and feasibility of the used telemedicine device, as well as the perceived helpfulness of the telemedicine support. Additionally, the remote consultant evaluated the utility of the device following the scenario with telemedicine support.

### Telemedicine Devices

- A conventional “audio-only” phone with headset (Engage 65 SE Mono headset, Jabra, Germany).
- Augmented reality glasses: the Microsoft HoloLens 2 (Microsoft, USA), used in this study in combination with the VSI HoloMedicine software (apoQlar, Hamburg, Germany), is a hands-free, gesture- and voice-controlled device equipped with a semitransparent head-mounted display and a central camera. It enables streaming of a first-person view, enhanced with holographic images. This allows the remote consultant to share the same view as the local team member. In the present study, only vital parameters derived from the patient monitor were displayed via augmented reality.
- A mobile video telemedicine device: this enables visualization of both the infant and the local team and has been considered useful for remote neonatal examinations [17, 18]. The Teladoc (Teladoc Lite v4 with Boom Arm, Teladoc Health, NY, USA) used in this study allowed the remote consultant to control three cameras on the local device through a software application on a conventional computer.

### Study Procedure and Scenario

To reflect common emergency situations encountered in hospitals providing basic perinatal care without on-site neonatology coverage, term newborn scenarios were selected. All participants performed the same two scenarios of comparable complexity involving the resuscitation of a term newborn after birth, based on suspected placental abruption and shoulder dystocia (online suppl. Material SM1). At the beginning of the simulation, participants received a short introduction about the available equipment. Participants were then asked to lead the simulated neonatal resuscitation scenarios, with a 15-min break in-between, according to their randomization. The neonatal resuscitation team consisted of the participant, a sim-midwife, a sim-nurse, and an additional team member who could be called for assistance and perform tasks such as catheter insertion when instructed. During the telemedicine scenario, participants initiated contact with the teleconsultant, who then provided support according to the capabilities of the assigned telemedicine device. The consultant was instructed to allow the participant to lead the resuscitation but offer guidance upon request or intervene with clear instructions if the participant failed to follow the guidelines or omitted necessary interventions. Afterward, the participants were asked to complete the questionnaires as described above. All questionnaires were provided in electronic form using SoSci Survey (SoSci Survey GmbH, Munich, Germany) on a tablet (Microsoft Surface, Microsoft, USA).

### Data Analysis

Statistical analyses were performed using R software (R Core Team, 2021). Descriptive statistics are presented as means and standard deviations. To account for the randomized crossover design and the resulting nonindependence of repeated observations within participants, NLS performance scores were analyzed using linear mixed-effects models with participant specified as a random intercept. Fixed effects included condition, defined either as no telemedicine versus telemedicine support or as no telemedicine, headset, HoloLens, and Teladoc. Randomization sequence was additionally examined as a fixed effect to assess potential order effects. Associations between participants’ level of training and performance were examined using linear mixed-effects models including condition, level of training, and their interaction as fixed effects, with participant specified as a random intercept; condition-specific slopes were estimated using marginal trends. Gain of performance scores, defined as the within-participant difference between telemedicine and no-telemedicine performance scores, were analyzed descriptively and correlated with level of training using Pearson’s r. Secondary participant-reported outcomes that were assessed once per participant or once per telemedicine condition were analyzed using linear models or one-way ANOVAs with Tukey-adjusted post hoc comparisons, as appropriate. Consultant ratings were reported descriptively only because all ratings were provided by a single remote consultant. Significance was set at *p* < 0.05.

## Results

### Study Group

Overall, 93 medical students and doctors participated in the study. Demographic data are shown in Table 1. The overall level of training, assessed using a composite training score (range 0–15), was 6.19 (SD = 4.62, median = 4) and did not differ between the three telemedicine support conditions (*F* = 0.394, *p* = 0.676).

**Table 1.** Demographic data of the study group

Characteristics	Participants, *n* (%)
Age	
20–30^a^	69 (74.2)
31–40^a^	17 (18.2)
41–50^a^	7 (7.5)
Gender	
Female	66 (70.9)
Profession	
Medical students	55 (59.1)
Residents Neonatology	18 (19.4)
Residents Anesthesiology	1 (1.1)
Consultants Neonatology	18 (19.4)
Consultants Anesthesiology	1 (1.1)
Experience score	
0–5 points	56 (60.2)
6–10 points	17 (18.3)
11–15 points	20 (21.5)

^a^ Years of age (annus).

### NLS Performance

When comparing the two conditions – with versus without telemedicine support – linear mixed-effects modelling with participant specified as a random intercept confirmed significantly higher externally assessed performance scores in the telemedicine condition, *F*(1, 93) = 166.17, *p* < 0.001. Estimated marginal means showed higher NLS performance with telemedicine support than without telemedicine support (M = 38.37, 95% CI [37.73, 39.01] vs. M = 33.02, 95% CI [32.38, 33.66]), corresponding to an estimated mean difference of 5.35 performance points. In addition, telemedicine support was associated with reduced variability in performance, as reflected by the smaller observed standard deviation in the telemedicine condition (Fig. 2). When examining the telemedicine conditions separately, linear mixed-effects modelling with participant specified as a random intercept showed a significant overall effect of condition, *F*(3, 139.11) = 55.64, *p* < 0.001. In a separate four-condition mixed model distinguishing the three telemedicine devices, estimated marginal means indicated that all telemedicine conditions were associated with significantly higher NLS performance than the no-telemedicine control condition (headset: *M* = 38.10, 95% CI [36.93, 39.26]; HoloLens: *M* = 38.49, 95% CI [37.42, 39.56]; Teladoc: *M* = 38.50, 95% CI [37.42, 39.59]; no telemedicine: *M* = 33.05, 95% CI [32.41, 33.69]; all *p* values <0.001). The three telemedicine conditions did not significantly differ from each other, indicating comparable performance across devices (*p* values ≥0.958). The level of training showed a significant effect on the performance scores in the scenario without telemedicine (slope = 0.29, 95% CI [0.15, 0.42]). No significant effects of the level of training were found for all three telemedicine conditions, including headset (slope = 0.06, 95% CI [−0.25, 0.38]), HoloLens (slope = −0.06, 95% CI [−0.26, 0.14]), and Teladoc (slope = −0.001, 95% CI [−0.23, 0.22]). This pattern is also evident when performance is stratified by experience groups (Fig. 2c). As a manipulation check, we used the same linear mixed-effects modelling approach to examine whether randomization sequence – no support first versus telemedicine support first – influenced performance. Neither the main effect of condition sequence, *F*(1, 93) = 0.91, *p* = 0.343, nor the condition × sequence interaction, *F*(1, 93) = 0.80, *p* = 0.374, reached significance, indicating that randomization order did not affect performance.

**Fig. 2.**
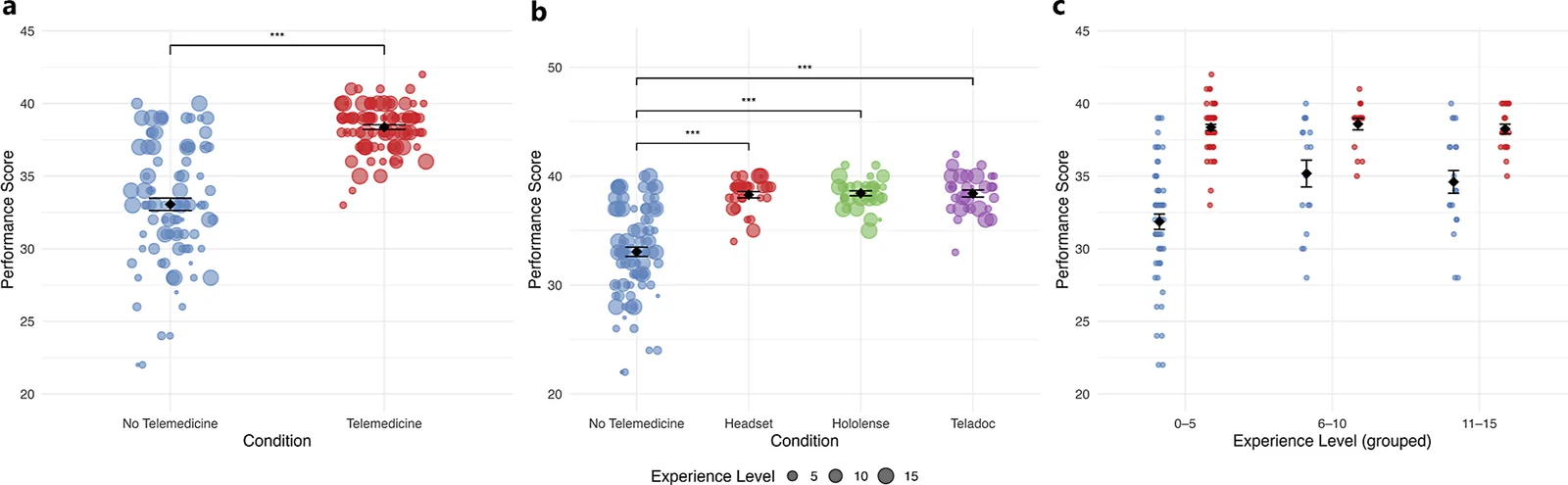
Comparison of performance score ratings. **a** Performance scores in no-telemedicine conditions versus telemedicine conditions. **b** Performance scores across individual telemedicine conditions. **c** Performance scores in no-telemedicine (blue circle) versus telemedicine (red circle) conditions stratified by experience level. Larger circles indicate higher participant level of training/experience level; circle size does not represent statistical weighting or overlapping observations.

### Gain of Performance by Using Telemedicine Support

In addition to the model-based comparison above, we calculated a descriptive “gain of performance” score as the difference between telemedicine and no-telemedicine performance. This gain score showed an average improvement of *M* = 5.32 points (SD = 4.03). Correlation analysis revealed that lower levels of training were associated with greater performance gains when telemedicine support was provided (*r* = −0.34, *p* < 0.001). Again, the telemedicine device used did not significantly influence the interaction between participants’ level of training and performance improvement (*p* > 0.716). Consequently, individuals with lower levels of training benefited from telemedicine support irrespective of the specific device used.

To further contextualize the clinical relevance of this performance gain, we conducted a descriptive component-level analysis of scored landmarks underlying the NLS performance score. Results suggested that the overall performance gain was mainly driven by better adherence to ventilation/airway management and reassessment/monitoring (Fig. 3).

**Fig. 3.**
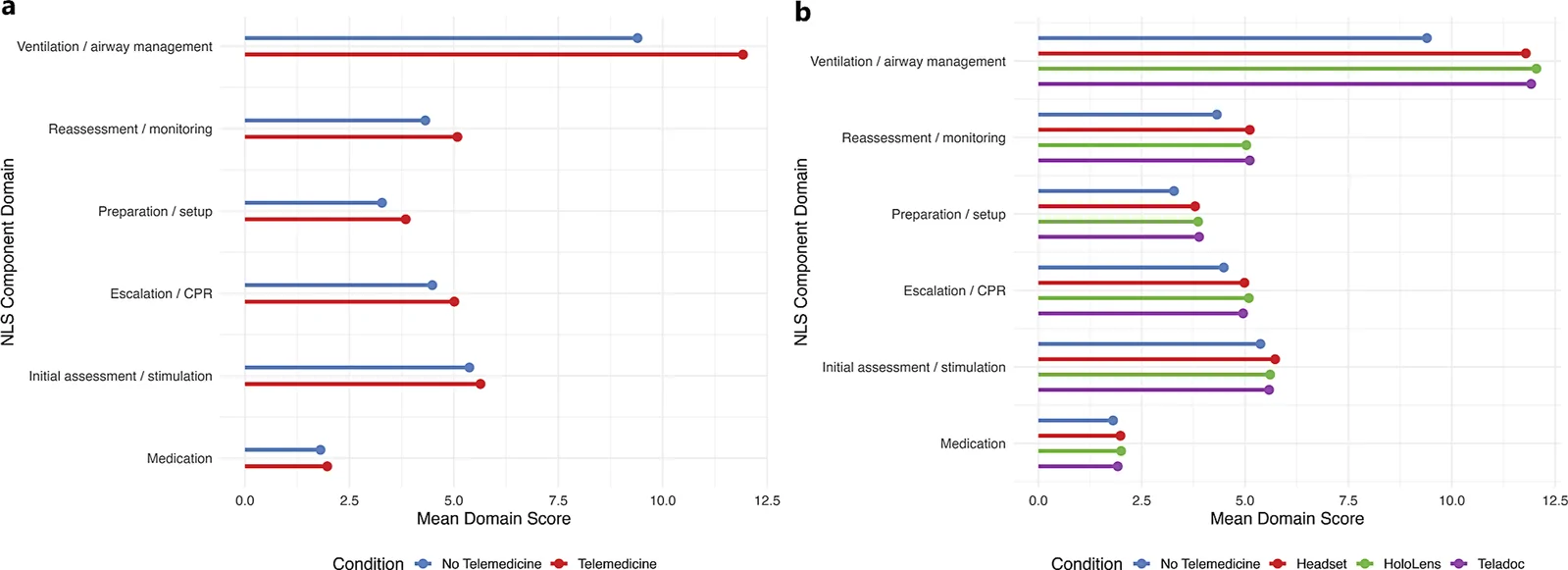
Component-level analysis. Descriptive component-level analysis of landmark components underlying the NLS performance score. **a** No-telemedicine condition versus telemedicine condition. **b** No-telemedicine condition versus individual telemedicine conditions.

### Participants’ Experience

When calculating a mean usability score based on the five items shown in Figure 4, the HoloLens received significantly lower ratings (M = 2.94, SD = 0.78) compared to both the headset (M = 3.49, SD = 0.49, *p* = 0.002) and Teladoc (M = 3.41, SD = 0.46, *p* = 0.007). No significant difference was observed between the headset and Teladoc (*p* = 0.842). The lower ratings for the HoloLens were primarily attributed to usability and technical issues, which were reported more frequently with this device. Descriptive consultant ratings are shown in online supplementary. Material SM 5 and 6.

**Fig. 4.**
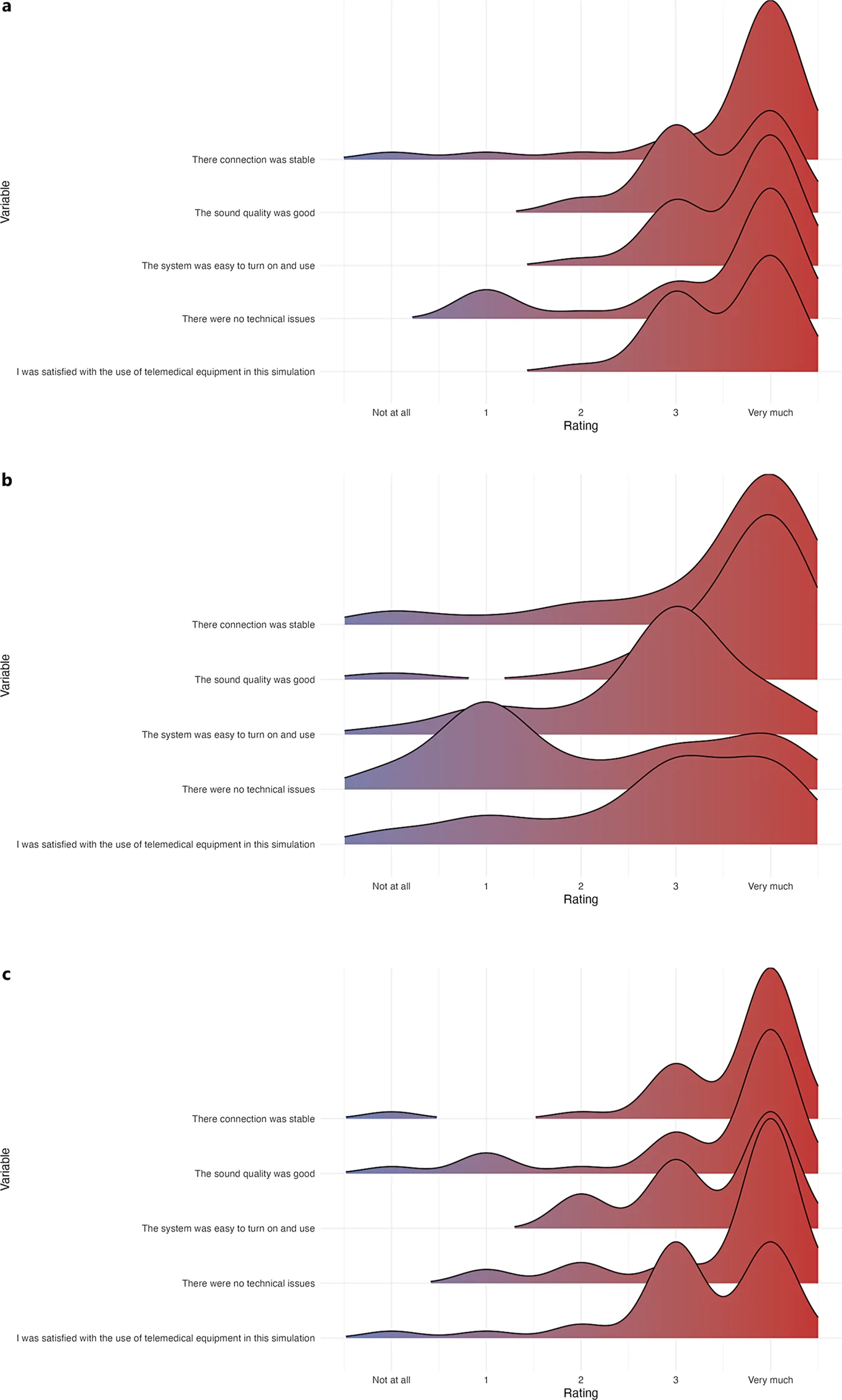
Participant ratings. User experience assessment of the three telemedicine conditions regarding usability: headset (**a**), augmented reality glasses HoloLens (**b**), mobile video telemedicine device Teladoc (**c**).

### Perceived Helpfulness of Telemedicine

Finally, participants’ subjective impressions regarding the helpfulness of telemedicine, potential distraction, and its perceived impact on simulation outcomes were assessed through a questionnaire. There were no significant differences between the three telemedicine conditions on any of these measures (Fig. 5). The level of training was negatively associated with perceived helpfulness (slope = −0.28, 95% CI [−0.45, −0.11]) and perceived improvement in the simulation (slope = −0.09, 95% CI [−0.13, −0.05]) but was not associated with perceived distraction (slope = 0.05, 95% CI [−0.01, 0.11]). Thus, participants with lower levels of training perceived telemedicine support as more helpful and reported greater improvement in simulation outcomes.

**Fig. 5.**
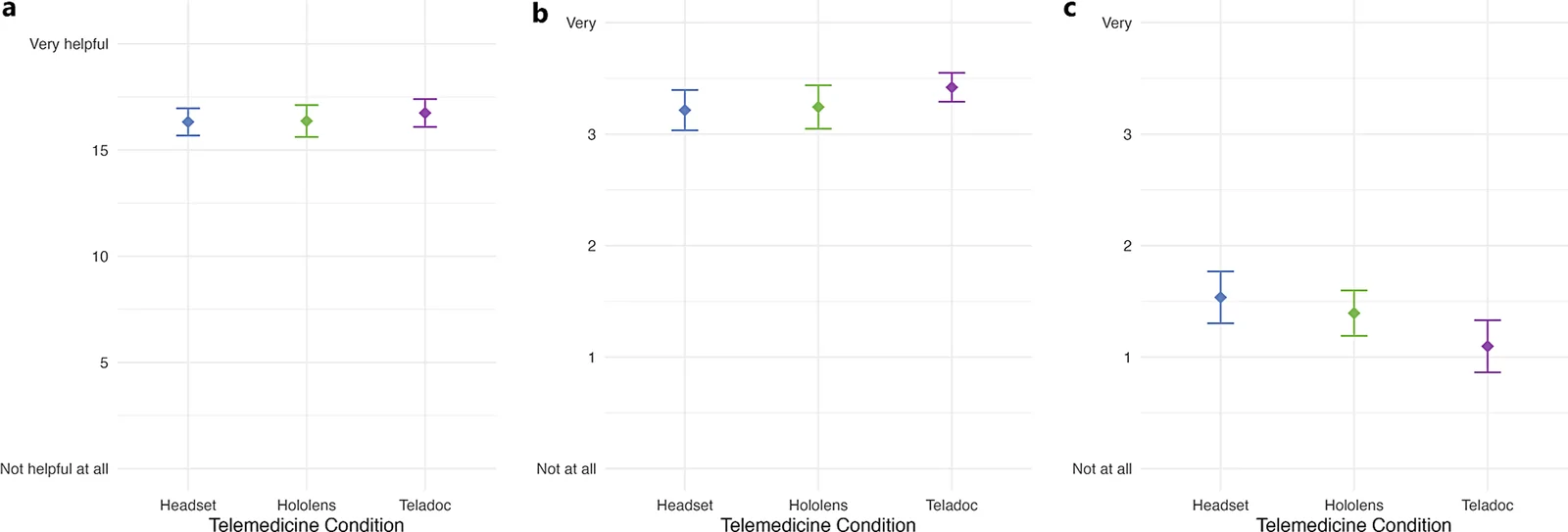
Additional self-report ratings for telemedicine conditions. **a** Perceived helpfulness of each condition. **b** Perceived improvement in outcome due to telemedicine. **c** Distraction caused by the telemedicine device.

## Discussion

In this randomized crossover simulation study, we evaluated the impact of telemedicine support on neonatal resuscitation performance and compared the usability and feasibility of three applicable devices. Our findings indicate that the use of telemedicine significantly improved participants’ performance scores across all three devices when compared to no telemedicine support. This highlights the potential of telemedicine to enhance clinical decision-making and execution, supporting previous research and guidelines that underscore the benefits of telemedicine consultation in critical care situations [8, 9, 11, 12, 18].

Importantly, the improved performance was evident specifically in less trained participants. In our study, participants’ level of training influenced performance only in the absence of telemedicine support. Although these findings are based on a simulation setting, they suggest that telemedicine could be especially valuable for bridging skill gaps in real clinical settings. Previous simulation-based and clinical studies have similarly shown that telemedicine can enhance outcomes during resuscitation conducted by less trained providers [11, 19–21].

Successful integration into clinical practice requires team acceptance and adequate resources [17]. In our study, participants in all telemedicine conditions perceived telemedicine support as helpful, indicating that the clinical value of telemedicine lies in strengthening collaboration, decision-making, and confidence among care teams. These aspects are particularly relevant in remote hospitals, where direct access to neonatal specialists is limited and telemedicine guidance can bridge critical expertise gaps. However, we observed notable differences in device usability. Reliability and immediate operability are essential for user confidence, as connectivity issues and complex setup during resuscitation remain significant barriers to clinical acceptance [9, 22]. In our study, the AR glasses were particularly associated with usability challenges, as setup was technically complex, which may limit their suitability in emergency situations. For less experienced participants, the AR-based display of vital parameters required continuous adjustment of viewing direction, which may have contributed to distraction during resuscitation. While the presence of telemedicine support is key, successful integration depends on technological feasibility and reliability. Issues like connectivity and complex interfaces must be mitigated by intuitive, high-quality systems designed for acute care [23]. However, device selection should also consider local conditions. While video telemedicine devices provide superior overview, head-mounted devices suit space-limited environments such as ambulances.

### Strengths and Limitations

The main strength of this study is its large and randomized design, including 93 participants with different levels of training. By assessing both performance and user experience, the study provides a comprehensive perspective on the impact of telemedicine on neonatal resuscitation.

A major limitation is the simulation setting itself: medical students represented the majority of participants and served as proxies for less experienced remote healthcare providers. The relatively young age of the study population and the predominance of participants with fewer years of training may not reflect the workforce structure in remote hospitals, where providers are often older and have more cumulative experience but limited exposure to neonatal resuscitation. Differences in baseline clinical competence, cognitive load, ability to integrate remote expert advice, and adaptability to technologies such as AR glasses may therefore limit generalizability of our findings and may also have influenced the observed effect size. In addition, the absence of a full interprofessional delivery room team (e.g., obstetricians, midwives, nursing staff) creates an artificial environment and should be addressed in further studies involving more representative clinical teams. The 15-min interval between simulation scenarios may also have been insufficient to fully eliminate potential carryover effects, such as cognitive fatigue, residual stress, or increased vigilance associated with repeated high-fidelity resuscitation simulations. Furthermore, the study did not include a preterm birth scenario, which represents an important clinical situation in remote settings and should be considered in future research.

To reduce bias between consultations, only one consultant was involved. This enhances comparability, however, the consultant’s prior knowledge of the simulation scenarios – despite clear instructions to minimize influence – may have introduced subtle biases. It may also limit the generalizability of the telemedicine device evaluation as it reflects a single observer’s perspective. Finally, the study design did not include assessment of a potential learning effect of the consultant over the course of the study.

## Conclusion

This simulation-based pilot study demonstrates that telemedicine support can enhance clinical performance in neonatal resuscitation, especially among less trained providers. While the specific type of telemedicine device did not significantly affect outcomes, the findings underscore that “simple-to-use” technology is needed by healthcare providers when using telemedicine.

## Statement of Ethics

The study protocol was approved by the Ethics Committee and the Data Protection Commission of Medical University of Vienna (EK No. 1686/2022). All participants provided written informed consent before participating in the study. Participants did not receive any compensation for participating in the study.

## Conflict of Interest Statement

The authors have no conflicts of interest to declare.

## Funding Sources

This study was funded by the “Digitalisierungsfonds” of the Chamber of Labour, Vienna, Austria (Grant No. FA781B3601). The funder had no role in the design, data collection, data analysis, and reporting of this study.

## Author Contributions

Dr. Lietz oversaw data collection, gathered the data, conducted the initial analyses, and prepared the first draft of the manuscript. Dr. Wagner conceptualized and designed the study and provided critical revisions to the manuscript. Dr. Goreis conducted the statistical analysis, managed data presentation, and contributed to the critical review and revision of the manuscript. Ms. Lair and Ms. Schedler supported and facilitated data collection efforts. Mr. Mahlberg programmed data collection instruments, collected data, and reviewed and revised the manuscript. Dr. Kothgassner and Dr. Berger supervised the project and provided critical reviews to ensure the manuscript's intellectual rigor. Dr. Rittenschober-Boehm conceptualized and designed the study, supervised data collection, obtained the necessary funding for the project, and provided critical revisions to the manuscript. All authors approved the final manuscript as submitted and agree to be accountable for all aspects of the work.

## Data Availability

The data that support the findings of this study are not publicly available due to confidentiality but are available from the corresponding author upon reasonable request.
